# Killer Cell Immunoglobulin-Like Receptors *(KIRs)* Genotype and Haplotype Analysis in Iranians with Non-Melanoma Skin Cancers

**DOI:** 10.29252/.23.5.330

**Published:** 2019-09

**Authors:** Fahimeh Yousefinejad, Farideh Jowkar, Shaghik Barani, Elham Jamali, Ebrahim Mahmoudi, Amin Ramezani, Elham Mahmoudi Maymand, Abbas Ghaderi

**Affiliations:** 1 Department of Immunology, School of Medicine, Shiraz University of Medical Sciences, Shiraz, Iran;; 2 Department of Dermatology, School of Medicine, Shiraz University of Medical Sciences, Shiraz, Iran;; 3 Shiraz Institute for Cancer Research, School of Medicine, Shiraz University of Medical Sciences, Shiraz, Iran;; 4 Cellular and Molecular Research Center, Basic Health Sciences Institute, Shahrekord University of Medical Sciences, Shahrekord, Iran;; 5 Department of Medical Biotechnology, School of Advanced Medical Sciences and Technologies, Shiraz University of Medical Sciences, Shiraz, Iran

**Keywords:** Basal cell carcinoma, KIR, Natural killer cells, Receptors, Squamous cell carcinoma

## Abstract

**Background:**

The innate immune system against malignancies is mainly orchestrated by natural killer cells, which carry out killing mechanisms by using their receptors, such as killer immunoglobulin-like receptors (KIRs). This study was designed to determine the diversity of KIR genes in non-melanoma skin cancers.

**Methods:**

A total of 160 subjects with skin cancer, including 60 cases of squamous cell carcinoma and 100 cases of basal cell carcinoma (BCC), and 270 healthy subjects formed the study groups. The sequence-specific polymerase chain reaction was carried out to detect the presence or absence of 16 *KIR *genes.

**Results:**

*KIR3DL1* (*p* = 0.0381, OR = 4.78, 95% CI = 1.108 to 20.62) increased in BCC patients compared to healthy controls.

**Conclusion:**

We concluded that the higher frequency of KIR3DL1 in BCC patients compared with healthy controls may increase the probability of developing BCC in Iranians.

## INTRODUCTION

Based on the WHO report, cancer is the second leading cause of mortality worldwide. Non-melanoma skin cancer is more prevalent in males compared to females in Iran^[^^1^^]^ although the probability of metastasis of non-melanoma types is less than melanoma one; non-melanoma types are more common^[^^2^^]^. Keratinocyte carcinomas or non-melanoma skin cancer include(s) several groups of skin cancers different in their etiopathogenesis, clinical course, and management strategies; however, it generally mentioned as “basal-cell carcinoma (BCC) and squamous-cell carcinoma (SCC)”, which is more aggressive in non-melanoma types^[^^3^^]^. The etiology of keratinocyte carcinomas, similar to other cancers, is multifactorial, which includes environmental factors such as long-term sun exposure^[^^4^^,^^5^^]^, human papillomavirus infection^[^^6^^]^, and host factors such as age^[^^7^^]^, skin pigmentation, genetic factors^[^^8^^]^, and the immune system status^[^^3^^]^. 

The impact of host genetic background on characteristics of skin cancer is highly important in a way that may lead to the change in behavior and cellular morphology; therefore, evaluation at the molecular level can be a tool for diagnosis and monitoring of the cancer. Human leukocyte antigen (HLA) has a key role in antigen presentation to T cells. Therefore, HLA I and II are among the molecules that are associated with susceptibility to different diseases like skin cancer^[^^9^^]^; for example, in one study it has been shown that the frequency of HLA-B27 and HLA-DR7 in patients with skin cancer is greater than in normal healthy controls^[^^10^^]^. Furthermore, the killer immunoglobulin-like receptor (KIR), a complex and diverse family of receptors expressing on natural killer (NK) cells, has similarly been studied for susceptibility or protection in various cancers^[^^11^^]^.

NK cells are composed of 5-15% of peripheral blood lymphocyte circulating at a different variable level in the liver and lung^[^^12^^]^. NK cells have a critical role in fighting against stressed, infected, as well as transformed cells^[^^13^^]^. NK cells can distinguish these cells by a collection of activating and inhibitory germline-encoded receptors^[^^14^^]^. Attainment of NK cell receptors, unlike T and B lymphocytes, is independent of gene arrangements. A certain ligation of HLA class I variants with inhibitory NK cell receptors inhibits NK cells from activation. NK cells act in response to diminishing HLA class I expression on target cells, which is called “Missing self”. Subsequently, the NK cells secret the interferon-gamma (IFN-γ) that increases HLA class I expression on tumoral cells and the HLA class II on antigen-presenting cells; consequently, triggering both arms of the immune responses^[^^15^^]^.

KIRs are the key receptors of NK cells^[^^16^^]^. A certain HLA class I is considered as KIR ligands categorizing three different epitopes, the Bw4, C1, and C2 epitopes, as well as HLA-A3/11 alleles. In particular, the recognition of Bw4, C1, and C2 epitopes by educated NK cells leads to subsequent interaction and destroy of the transformed target cells^[^^17^^,^^18^^]^. The high diversity of *KIR* gene family is determined by variable gene content in haplotypes and allelic polymorphism^[^^19^^]^. Among 16 *KIR *genes, *KIR3DL3*, *3DL2*, *2DL4*, and *3DP1*, as framework genes, exist in approximately all individuals. The KIR haplotypes are divided into A and B groups based on their gene content^[^^20^^]^. Group A haplotypes have specified gene contents (*KIR3DL3, 2DL3, 2DP1, 2DL1, 3DP1, 2DL4, 3DL1, 2DS4, *and *3DL2*). In contrast, group B haplotypes have many variable genes (*KIR2DL2-2DL5-2DS1-2DS2-2DS3-2DS5-3DS1*) that did do not occur in A haplotype. To find out the impact of *KIR* genes content on the risk of non-melanoma skin cancers development, the *KIR* typing was performed in 160 patients and 270 healthy controls. 

## MATRIALS AND METHODS

**Study population**

The patient group was consisted of 160 non-melanoma skin cancer, 100 BCC and 60 SCC which were compared to 270 healthy controls from Fars Province of Iran. The patients and healthy controls were age-sex matched with negative family history of cancer (Table 1). The cases were recruited at Shahid Faghihi Hospital, Shiraz, Iran. The research was approved by the Medical Research Ethics Committee in Shiraz University of Medical Sciences (EC-9376-7306). 

**KIR genotyping**

DNA of EDTA-treated blood samples were extracted using Qiagen QIAamp DNA Blood Mini Kit (Germany). We used a sequence-specific polymerase chain reaction (SSP-PCR) method with certain PCR conditions for detection of *KIR* genes, which was previously described by Barani *et al.*^[^^21^^]^. PCR was performed using Eurofin Genomics primers (Ebersberg, Germany). Subsequently, 5 µl of PCR products were electrophoresed on 2% agarose gels and detected by a UV transilluminator. The panel of 10 UCLA KIR exchange DNA was applied to confirm specificity and accuracy of the test^[^^22^^]^. 

**Statistical analysis**

We used direct counting to determine the frequency of each gene, haplotype and Bx subsets, and gene clusters. The frequencies in patients compared with healthy controls were calculated for two-tailed Chi-square with Yates’ correction, and *p* < 0.05 was considered to be statistically significant. Odds ratio and the 95% confidence intervals were reported as well. Based on linkage disequilibrium in the *KIR *system, two clusters, defined as C4 and T4, were characterized at the telomeric and centromeric half^[^^23^^,^^24^^]^ and calculated as described previously^[^^25^^]^.

## RESULTS

**KIRs gene content diversity in BCC and SCC patients**

We studied 430 individuals who represented BCC and SCC patients as well as healthy controls to find out the impact of *KIR* genes content on the risk of developing non-melanoma skin cancers. The framework genes existed in all the individuals. We observed a significantly higher frequency of *KIR3DL1* in the BCC compared to the healthy controls (98% vs. 91.1%, *p* = 0.0381, OR = 4.78, 95% CI = 1.108 to 20.62) but was not significant, based on adjusted *p* value <0.025 after Bonferroni correction. However, in the comparison between the SCC and controls, we did not observe any significant difference (Table 2), but we observed 40 distinct KIR genotypes in the study population. There were 10 unique genotypes (Table 3), which were only present in one individual, (five in patients and five in healthy controls). We rechecked and confirmed unique genotypes by different sets of primers designed by Ashouri *et al.*^[^^25^^]^.

**Table 1:** Gender distribution of the cases and controls

**Gender**	**BCC** **(n = 100)**	** SCC** ** (n = 60)**	**Controls** **(n = 270)**
Male % (n)	39 (39)	26.6 (16)	29.3 (79)
Female % (n)	61 (61)	73.4 (44)	70.7 (191)
Mean age (SD)	61 (17)	66.0 (15)	63.0 (12)

***KIR2DS4*** **variants distribution in BCC and SCC patients**

Different variants of *KIR2DS4* have previously been determined in many research studies^[^^21^^,^^26^^-^^28^^]^. To study the frequency of *KIR2DS4 *variants, we categorized the study population into three groups: (A)* 2DS4Del:* deleted variant of *KIR2DS4*, (B)* 2DS4Full: *full-length *KIR2DS4*, and (C)* 2DS4Full/Del: *full-length and deleted variants of KIR2DS4. Only one variant of 2DS4 (2DS4*001 or full length) encodes a functional protein acting as a membrane receptor. By investigating the possibility of any significant relationship between the disease and variants of *2DS4*, we observed that the full length of *2DS4* was significantly higher in the BCC patients compared to the controls (*p* = 0.0415, OR = 2.01, 95% CI = 1.074 to 3.781) but was not significant after Bonferroni correction, we, however, found no significant difference between the patients with SCC and the controls (Table 4).

**The frequency of AA and Bx genotypes**

The frequency of AA genotype (genotype #1), the homozygote combination of A haplotypes, occurred in higher frequency in the cases compared to the controls, but this difference was not statistically significant. 

**Table 2:** Frequency of *KIR* genes in BCC, SCC, and healthy controls

***KIR *** **gene**	**BCC** **(n = 100)**	**SCC** **(n = 60)**	**Controls (n = 270)**		**BCC vs. control**				**SCC vs. control**		
	**%F (N+)**	**%F (N+)**	**%F (N+)**		***p*** ** value**	**OR**	**%95 CI**		***p*** ** value**	**OR**	**95**% **CI**
**A haplotypes associated-** ***KIR *** **genes**											
*2DL1*	100.0 (100)	100.0 (60)	100 (270)		_	NA			_	NA	
*2DL3*	86.0 (86)	90.0 (54)	89.2 (241)		0.36	0.73	0.37-1.46		1.00	1.08	0.42-2.73
*3DL1*	98.0 (98)	90.0 (54)	91.1 (246)		**0.0381** * †	**4.78**	**1.10-20.6**		0.80	0.87	0.34-2.25
*2DS4*	98.0 (98)	93.3 (56)	91.8 (248)		0.0581	4.34	1.00-18.84		1.00	1.24	0.41-3.74
**B haplotypes associated-** ***KIR*** ** genes**											
*2DL2*	62.0 (62)	61.6 (37)	65.9 (178)		0.54	0.84	0.52-1.35		0.55	0.83	0.46-1.48
*2DL5*	64.0 (64)	66.6 (40)	62.9 (170)		0.90	1.04	0.64-1.68		0.65	1.17	0.65-2.12
*3DS1*	42.0 (42)	36.6 (22)	37.7 (102)		0.47	1.19	0.74-1.90		0.11	0.62	0.36-1.08
*2DS1*	42.0 (42)	36.6 (22)	45.9 (124)		0.55	0.85	0.53-1.35		0.19	0.68	0.38-1.21
*2DS2*	61.0 (61)	65.0 (39)	61.8 (167)		0.28	1.30	0.81-2.09		0.76	1.14	0.63-2.05
*2DS3*	51.0 (51)	50.0 (30)	45.1 (122)		0.34	1.26	0.79-1.99		0.56	1.21	0.69-2.12
*2DS5*	28.0 (28)	31.6 (19)	32.9 (89)		0.38	0.79	0.47-1.31		0.88	0.94	0.51-1.71
**Framework genes/pseudogenes**											
*2DL4*	100 (100)	100 (60)	100 (270)		_	NA			_	NA	
*3DL2*	100 (100)	100 (60)	100 (270)		_	NA			_	NA	
*3DL3*	100 (100)	100 (60)	100 (270)		_	NA			_	NA	
*2DP1*	100 (100)	100 (60)	99.6 (269)		1.00	1.12	0.04-27.7		1.00	0.67	0.02-16.75
*3DP1*	100 (100)	100 (60)	100 (270)	_		NA		_		NA	

Frequency of KIR genes was expressed as the percentage and defined as the number of individuals with KIR genes (N+) divided by a number of individuals studies in the given study group (n). Two-tailed Fisher's exact probability (p) test, Odd ratio with 95% CI, was calculated by Graphpad Prism software, and

* *p* < 0.05 was considered statistically significant, based on two-tailed Chi-square with Yates' correction. NA, not available;

† *p* < 0.025, statistically non-significant, based on Bonferroni correction

**Table 3:** KIR genotypes distribution in the study population

**# Genotype**	**KIR ID**	**Group**	**3DL1**	**2DL1**	**2DL3**	**2DS4**	**2DL2**	**2DL5**	**3DS1**	**2DS1**	**2DS2**	**2DS3**	**2DS5**	**2DL4**	**3DL2**	**3DL3**	**2DP1**	**3DP1**	**BCC ** **(n = 100)**			**SCC ** **(n = 60)**			**Control ** **(n = 270)**	
																			F% (N+)	F% (N+)		F% (N+)	F% (N+)		F% (N+)	F% (N+)
1	1	AA																	23.0	23		23.33	14		20	54
2	14	Bx																	1.0	1					0.74	2
3	23																								0.37	1
4	200																		1.0	1					0.74	2
5	2																		9.0	9		11.66	7		6.66	18
6	27																		1.0	1					0.74	2
7	8																		3.0	3						
8	10																					1.66	1			
9	12																					1.66	1		0.37	1
10	19																								3.33	9
11	18																								1.11	3
12	58																		1.0	1						
13	4																		11.0	11		8.33	5		11.85	32
14	31																		1.0	1		1.66	1		1.11	3
15	9																								1.85	5
16	3																		3.0	3		1.66	1		3.33	9
17	5																		16.0	16		20.00	12		8.51	23
18	13																					1.66	1		1.48	4
19	11																		1.0	1		1.66	1		4.81	13
20	7																		5.0	5		1.66	1		4.44	12
21	382																								2.22	6
22	6																		8.0	8		8.33	5		8.14	22
23	144																								0.37	1
24	190																					1.66	1		0.37	1
25	81																								0.74	2
26	297																					1.66	1			
27	180																		1.0	1						
28	317																								0.74	2
29	71																		3.0	3		6.66	4		4.44	12
30	113																		1.0	1					2.22	6
31	90																		4.0	4					0.74	2
32	73																		5.0	5					1.11	3
33	308																								0.37	1
34	69																								2.22	6
35	117																								0.37	1
36	75																		1.0	1					1.48	4
37	68																		1.0	1						
38	70																					5.00	3		2.22	6
39	86																								0.37	1
40	87																					1.66	1		0.37	1

The presence and absence of a KIR gene are indicated by a shaded and white box, respectively. KIR ID was assigned by the Allele Frequency Net Database.

**Table 4:** Distribution of *KIR2DS4* variants in the study population

***KIR2DS4 ***	**BCC ** **(n = 98)**	**SCC ** **(n = 56)**	**Control** **(n = 248)**		**BCC vs. control**				**SCC vs. control**		
	**%F (N+)**	**%F (N+)**	**%F (N+)**		***p*** ** value **	**OR**	**95** **%** ** CI**		***p*** ** value **	**OR**	**95** **%** ** CI**
Del	66.3 (65)	69.6 (39)	67.3 (167)		0.89	0.95	0.58-1.56		0.87	1.11	0.59-2.08
Full	20.4 (20)	14.3 (8)	11.3 (28)		**0.0415** * †	**2.01**	**1.07-3.78**		0.49	1.31	0.59-2.09
Full/Del	12.2 (12)	14.3 (8)	19.7 (49)		0.11	0.56	0.28-1.19		0.44	0.67	0.59-2.10
Missing	1.02 (1)	1.78 (1)	1.6 (4)		1.00	0.62	0.06-5.7		1.00	0.90	0.59-2.11

The number (n) that is exhibited below each group is the number of people who were 2DS4 positive in each group, which is categorized into three types (Deletion, Full, and Full/Deletion) of 2DS4 allele. Two-tailed Fisher's exact probability (p) test, odd ratio with KI95% CI was calculated by Graphpad Prism software;

* *p* < 0.05, statistically significant based on two-tailed Chi-square with Yates' correction;

† *p* < 0.025, statistically non-significant based on Bonferroni correction.

Consequently, the frequencies of Bx genotypes were not significantly different between the cases and healthy controls (Table 5). Also, no significant difference was found in the carriers of C4 and T4 clusters between the cases and controls (Table 6). 

## DISCUSSION

The high prevalence of cancers has been observed in individuals with defective NK cell functions^[^^29^^]^. A long-term epidemiological study has reported that individuals with low NK cell activity are prone to high risk of developing different types of cancers^[^^30^^]^. In this study, almost all of the BCC patients were carriers of *KIR3DL1*, a receptor binding to HLA-Bw4 epitope to transfer the inhibitory signal to NK cells. KIR3DL1 is a highly polymorphic allele (https://www.ebi.ac.uk /ipd/kir/ stats.html) with different spectra of cell surface expression. In consistency with this study, the combination of KIR3DL1 with less effective Bw4^T80 ^has been reported in primary melanoma patients compared to metastatic patients^[^^31^^]^, since KIR3DL1 generates a weaker inhibitory signal when an threonine residue is present at position 80 (Bw4T80). In contrast, the Bw4I80 epitope with an isoleucine at position 80 sends stronger inhibitory signals to NK cells^[^^32^^]^. Hence, the importance of investigating KIR receptors and HLA-KIR combinations in addition to KIR typing should be highlighted. It has previously been shown that the high frequency of *KIR3DL1 *in BCC with more activating KIR receptors could help to kill tumor cells with low/absence of HLA class I expression^[^^33^^]^. De Re *et al.*^[^^34^^]^ have reported that the presence of activating telomeric KIR receptors in the absence of KIR2DS4 and KIR3DL1 might increase the likelihood of complete response to chemotherapy in metastatic colorectal cancer patients. However, inhibitory KIRs are expressed on a group of effector/memory CD4+ and CD8+ T cells along with NK cells, and as a result, the inhibitory KIRs might decrease the antitumor responses of these cells. These data support a scheme that full competent NK cells with less activating KIR receptors may help to develop BCC cancer in some individuals.

**Table 5:** Frequency of KIR genotypes and haplotypes in BCC and SCC patients and healthy controls

** KIR genotypes and haplotypes**		**BCC ** **(n = 100)**	**SCC ** **(n = 60)**	**Control** **(n = 270)**		**BCC vs. control**				**SCC vs. control**		
		**%F (N+)**	**%F (N+)**	**%F (N+)**		***p*** ** value **	**OR**	**95** **%** ** CI**		***p*** ** value **	**OR**	**95** **%** ** CI**
Genotype	AA	23.0 (23)	23.3 (14)	20.0 (54)		0.501	1.26	0.72-2.19		0.69	1.21	0.62-2.37
	Bx	77.0 (77)	76.6 (46)	80.0 (216)								
												
Haplotype	A	53.5 (107)	53.0 (64)	50.9 (275)		0.97	1.01	0.64-1.58		0.63	1.10	0.74-1.63
	B	46.5 (93)	47.0 (56)	49.1 (265)								

Frequency of KIR genotypes/haplotypes was expressed as percentage and defined as the number of individuals with genotype/haplotype (N+) divided by the number of individuals studies in the given study group (n). No significant *p* value was found.

**Table 6:** Frequency of KIR Bx genotypes and KIR clusters in BCC and SCC patients and healthy controls

**Bx genotypes and KIR clusters**	**BCC ** **(n = 100)**	**SCC ** **(n = 60)**	**Control** **(n = 270)**		**BCC vs. control**				**SCC vs. control**		
	**%F (N+)**	**%F (N+)**	**%F (N+)**		***p*** ** value**	**OR**	**95** **%** ** CI**		***p*** ** value**	**OR**	**95** **%** ** CI**
C4Tx genotype	30.0 (30)	33.3 (20)	29.2 (79)		0.89	1.03	0.62-1.71		0.53	1.20	0.662.19
CxT4 genotype	15.0 (15)	15.0 (9)	15.9 (43)		0.87	0.93	0.49-1.76		1.00	0.93	0.42-2.03
C4T4 genotype	13.0 (13)	16.6 (10)	12.6 (34)		1.00	1.03	0.52-2.05		0.40	1.38	0.64-2.99
CxTx genotype	19.0 (19)	11.6 (7)	22.2 (60)		0.56	0.82	0.46-1.46		0.07	0.46	0.19-1.07
C4 gene cluster	43.0 (43)	50.0 (30)	41.8(113)		0.91	1.03	0.69-1.53		0.32	1.26	0.79-2.00
T4 gene cluster	28.0 (28)	31.6 (19)	28.5 (77)		1.00	0.97	0.61-1.56		0.66	1.13	0.65-1.95

Two-tailed Fisher's exact probability (p) test was calculated by Graphpad Prism software, and *p* < 0.05 was considered to be statistically significant. No significant *p* value was found.

Higher frequency of *KIR2DS4 *full length carriers in BCC group is a clue showing the importance of inflammation in development and persistence of tumors. The *2DS4* is an activating KIR that can activate NK cells, resulting in the secretion of IFN-γ, a proinflammatory cytokine with anti-tumor property that regulates the transcription of many immune response genes^[^^35^^]^. These events and activation of NK cells occur when 2DS4 is in the full length because all its other variants are non-functional. A number of confirmatory studies have shown that KIR2DS4*001 (the full variant of *2DS4*) is associated with a relatively high viral load^[^^36^^,^^37^^]^ and transmission of HIV-1^[38]^. Consistent with these findings, Bao *et al.*^[^^39^^]^ in their research have found an association between KIR2DS4*001 and acute graft-versus-host disease. In contrast, Barani *et al.*^[^^21^^]^ did not find any association between *KIR2DS4 *full length and patients with head and neck SCC but showed a protective role of *KIR2DS4 Del *against disease. 

In the present study, we did not find any significant difference in the carrier frequencies of AA, Bx genotypes, A and B haplogroups, as well as Bx clusters between the patients (SCC and BCC) and controls, while AA genotype resistance and CxT4 genotype susceptibility have been reported in SCC patients with head and neck cancer in Iranians^[21]^. The results of this study, as shown in Table 4, was consistent with the a previous study, “KIR gene content diversity in Iranian populations”, supporting that the frequency of A haplotype was greater than B haplotype in Iranians (50.9% A haplotype vs. 49.1% B haplotype in healthy control group)^[^^25^^]^. That study also concluded that the AB genotype had higher frequency than BB genotype in Iranian populations, being consistent with our results in which the frequency of AB genotype was 61.8%, while the BB genotype was 18.2% in the healthy controls (data not shown). Future studies of the KIRs-HLA class I pattern in patients with non-melanoma skin cancer as well as γδ T cells, which have extent distribution in skin^[^^40^^]^ and functional studies of NK cells possessing KIR3DL1 in BCC patients, would be a valuable strategy to find out the effect of its presence on the NK cell meddling in cancer immunity. 
